# Polluted Air from Canadian Wildfires and Cardiopulmonary Disease in the Eastern US

**DOI:** 10.1001/jamanetworkopen.2024.50759

**Published:** 2024-12-13

**Authors:** Mary E. Maldarelli, Hyeonjin Song, Clayton H. Brown, Madhurika Situt, Colleen Reilly, Anup A. Mahurkar, Victor Felix, Jonathan Crabtree, Evan Ellicott, Martha O. Jurczak, Binod Pant, Abba Gumel, Zafar Zafari, Warren D’Souza, Amir Sapkota, Bradley A. Maron

**Affiliations:** 1 The University of Maryland-Institute for Health Computing, Bethesda; 2 Department of Epidemiology and Biostatics, University of Maryland School of Public Health, College Park; 3 The University of Maryland Medical System, Baltimore; 4 Department of Geographical Science, University of Maryland, College Park; 5 Department of Mathematics, University of Maryland, College Park; 6 Department of Practice, Sciences, and Health Outcomes Research at the University of Maryland School of Pharmacy, Baltimore

## Abstract

**Importance:**

Intense wildfires affecting residential populations are increasingly frequent. However, the adverse cardiopulmonary consequences to patients from remote wildfire smoke exposure is uncertain.

**Objective:**

To investigate the association between wildfire smoke originating in Western Canadian provinces with cardiopulmonary disease burden in sociodemographically heterogenous populations in the Eastern US.

**Design, Setting, and Participants:**

This case-only study used *International Statistical Classification of Diseases and Related Health Problems, Tenth Revision* (*ICD-10*) codes for cardiopulmonary diseases extracted from the University of Maryland Medical System in June 2023 vs June 2018 and June 2019. Data were analyzed from September 2023 to September 2024.

**Exposures:**

High air pollution episodes where the concentration of particulate matter with aerodynamic diameter below 2.5 μm (PM_2.5_) exceeded the toxic National Ambient Air Quality Standard (35 μg/m^3^) (referred to as “hotspot days”) on contiguous days.

**Main Outcomes and Measures:**

The number of patients with inpatient, ambulatory, and emergency department clinical encounters during assigned hotspot days in June 2023 compared with matching days in June of control years. Adjustments for covariates for comparisons between groups were made with χ^2^ tests and multivariable logistic regression.

**Results:**

Statewide air quality analysis identified June 6-8 and 28-30 as 6 hotspot days with an increase in PM_2.5_ by 9.4-fold and 7.4-fold, respectively, in Baltimore City compared with all other days in 2023. After adjusting for calendar days across years, the cohort included 2339 cardiopulmonary clinical encounters in June 2023 (mean [SD] age, 68 [15] years; 1098 female [46.9%]; 710 Black [30.4%], 1528 White [65.3%]) and 3609 encounters in June 2018-2019 (mean [SD] age, 65 [15] years; 1690 female [46.8%]; 1181 Black [32.7%], 2269 White [62.9%]). The proportion of clinical encounters occurring during hotspot days in June 2023 was 588 of 2339 days (25.1%) vs 806 of 3609 days (22.3%) in control years (*χ^2^* = 6.07; *P* = .01), with an adjusted odds ratio (aOR) of 1.18 (95% CI, 1.03-1.34; *P* = .02). Restricting this analysis to cardiac diseases, there was a 20% increase in adjusted odds for a clinical encounter (aOR, 1.20; 95% CI, 1.01-1.42; *P* = .04). Patients with cardiopulmonary encounters on hotspot days had greater socioeconomic advantage vs control years by ADI score (mean [SD] score, 39.1 [21.1] vs 41.0 [23.7]; *P* = .05).

**Conclusions and Relevance:**

In this case-only study of a large medical system, we identified an increased cardiopulmonary disease burden for residents of Maryland that was likely associated with contemporaneous wildfire smoke–based infiltration of polluted or toxic air originating from Western Canada up to 2100 miles remotely.

## Key Points

**Question:**

Is there an association between the infiltration of wildfire smoke originating from Western Canada and cardiopulmonary disease burden remotely in Maryland?

**Findings:**

In this case-only study, we used satellite and Environmental Protection Agency data to identify 6 calendar days in June 2023 with high levels of wildfire smoke–related air pollution. In multivariable analyses, these days demonstrated a significant increase in ambulatory clinic, inpatient, or emergency department clinical encounters for cardiopulmonary disease across a large, sociodemographically diverse medical system in Maryland.

**Meaning:**

These results suggest that adverse cardiopulmonary disease events in Eastern US urban and rural communities were associated with exposure to remote wildfire smoke.

## Teaser

This case-control study compares rates of clinical encounters for cardiopulmonary disease in the Eastern US following wildfires in Western Canada with previous periods of high air pollution within the same medical system.

## Introduction

Wildfires arising largely in rural sparsely populated areas of the Western US and Canada are increasing in size and intensity.^1^ One study from 2018^2^ estimated that 2500 deaths within states experiencing a wildfire were attributable to wildfire smoke exposure, and wildfire smoke is associated with exacerbation of cardiac disease, including coronary artery disease, in populations residing within a region near the wildfire origin.^3,4^ Similarly, acute wildfire smoke exposure has been implicated as a disease modifier of highly prevalent chronic conditions such as rhinosinusitis, asthma, and chronic obstructive pulmonary disease.^5,6,7^ However, while wildfire events are known to adversely affect the health and safety of local residential populations in these regions, it is not generally recognized that polluted air from wildfire smoke can adversely affect the cardiopulmonary health of patients at remote distances from the triggering events.

In June of 2023, Canada experienced a record-breaking wildfire season, with an estimated 6551 fires destroying 18.5 million hectares of forested land.^8^ The smoke from these fires contained numerous air pollutants, including particulate matter with aerodynamic diameter smaller than 2.5 μm (PM_2.5_), which were carried by atmospheric systems to the Eastern US, including Maryland, which is approximately 3400 km (2100 miles) distant, affecting air quality far beyond Canada’s borders.^9^ In this report, we describe in detail the clinical profile and transcontinental consequences of Canadian wildfire smoke events in 2023, as documented by a unique hospital network throughout Maryland.

## Methods

### Clinical Data

This case-only study included a multicenter, retrospective cohort of patients aged 18 years or older who presented to an ambulatory clinic, emergency department, and in-patient settings in June 2023 (case group) and June 2018 and June 2019 (control group) throughout the University of Maryland Medical System (UMMS), which included clinical operations in 9 counties across Maryland (eMethods in Supplement 1). This design was considered case-only because we included only patients with the diseases of interest in our exposed and unexposed cohorts, which differs from a traditional case-control design that would have also included individuals without the diseases of interest in the study cohorts.

The counties included in this study were: Baltimore, Harford, Anne Arundel, Prince Georges, Charles, Kent, Queen Anne’s, Talbot, Dorchester. Patients were extracted from the UMMS electronic health record using *International Statistical Classification of Diseases and Related Health Problems, Tenth Revision (ICD-10)* codes corresponding to cardiopulmonary diseases; ie, cardiac–nonheart failure, cardiac–heart failure, and respiratory (eTable 1 in Supplement 1). Therefore, in total, all patients ages 18 years and older experiencing a clinical encounter for any disease classified as cardiopulmonary during June 2018, 2019, and 2023 were included in this study. There were no exclusion criteria. Race and ethnicity were derived from the electronic health record, as determined by patient self-report and documented by staff; categories included Asian, Black or African American, White, or other (American Indian or Alaskan Native, Native American or other Pacific Islander, other, unknown, and declined to answer). Race was considered as a covariate, as previous studies have demonstrated pollution from wildfire smoke may have an adverse effect on persons who may identify with a racial minority group.^24^ A summary of missingness for each clinical variable is provided in eTable 2 in Supplement 1. The study was approved by the University of Maryland institutional review board with the requirement for informed consent waived due to minimal risk and the use of deidentified data in this study. This study follows the Strengthening the Reporting of Observational Studies in Epidemiology (STROBE) reporting guideline for case-control studies.

### Estimating Wildfire-Related PM_2.5_

We quantified census tract–level wildfire exposure using 2 metrics: wildfire smoke plume, and wildfire-related PM_2.5_ concentration, as previously described.^10,11,12^ In brief, daily atmospheric trends of PM_2.5_ were analyzed and compared with the Environmental Protection Agency-National Ambient Air Quality Standard (EPA-NAAQS) for PM_2.5_ (above 35 μg/m^3^) in each of the 23 counties in Maryland for 2018, 2019, and 2023 (2020-2022 were excluded due to the COVID-19 pandemic). Days exceeding this standard were designated as calendar “hotspot” days. Further details regarding the calculation of wildfire-related PM_2.5_ are provided in the eMethods in Supplement 1. Wildfire score was calculated per census tract in Maryland quantifying impact of wildfire smoke defined by: number of wildfire smoke days in June 2023 multiplied by wildfire smoke intensity (determined by satellite image opacity in each region).

### Statistical Analysis

The proportion of cardiopulmonary clinical encounters in June 2023 that occurred on hotspot days was analyzed. This value was compared with the proportion of clinical encounters that occurred on orthologous hotspot days in June 2018 and June 2019 combined, which acted as control years. To standardize days of the week across years, we compared the 4 complete weeks of June in each year starting with the first Monday and ending with the last Friday of the month. The hotspot days occurred on Tuesday through Thursday of week 1 and Wednesday through Friday of week 4 in June 2023 (and, thus, these were assigned as orthologous days in June 2018 and June 2019). Pearson χ^2^ test was used to compare the clinical encounter proportions between time periods (2023 vs 2018 + 2019). Multivariable logistic regression was used to adjust for covariates. Summary statistics for continuous data are presented as mean and median values for normally and nonnormally distributed data, respectively; *P* < .05 was considered statistically significant. All statistical tests were 2-sided and performed using R version 1.3.3 (R Project for Statistical Computing) with no specialized statistical analysis extension packages.

## Results

### Air Pollution

In June 2023 there were numerous days in which air pollution was clearly visible to residents of Baltimore City (Figure, A).^9^ Observations related to atmospheric trends in June 2023 indicated that the migration of a Canadian wildfire smoke plume coursed approximately 4000 km from Western Canada to Baltimore City (Figure, B; eFigure in Supplement 1), which was consistent with elevated wildfire scores for each of the 9 UMMS counties with clinical data available for this analysis (Figure, C). We identified 2 distinct periods in 2023 (June 6-8; 28-30) in which PM_2.5_ levels exceeded the NAAQS for 25% or more counties (ie, designated as hotspot days). During these hotspot days, there was an abrupt 9.4-fold (mean [SD], 70.8 [39.3] μg/m^3^ vs 7.5 4.6 μg/m^3^) (for June 6-8) and 7.4-fold (mean [SD], 55.8 [32.2] μg/m^3^ vs 7.5 [4.6] μg/m^3^) (for June 28-30) increase in PM_2.5_ concentration compared with all other days in 2023 (Figure, D).

**Figure.**
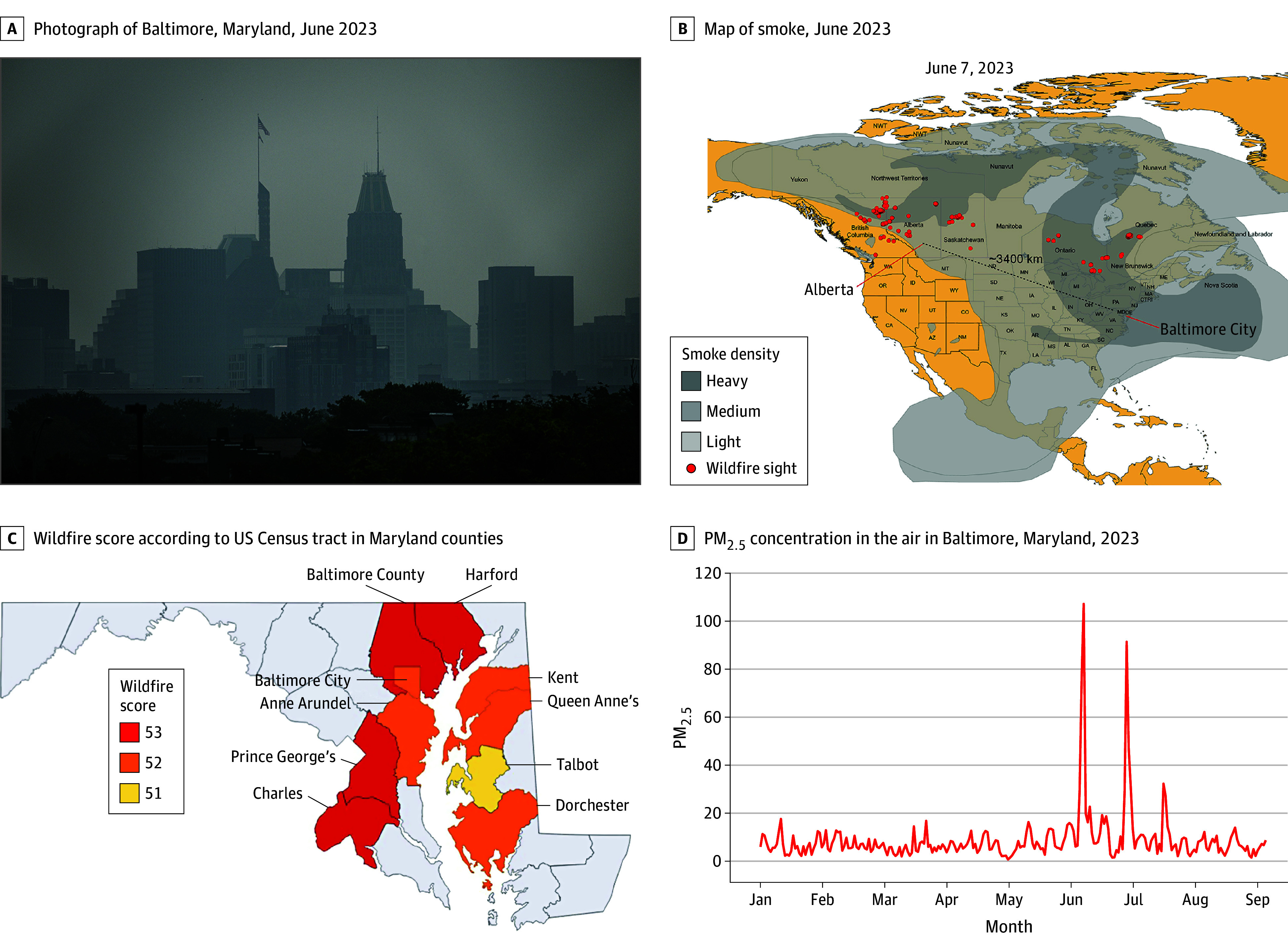
Satellite and Atmospheric Data Characterizing the Distribution of Toxic Wildfire Smoke Pollution Observed in Maryland, Originating From Western Canada, June 2023 A, A change in air quality was visually evident in Baltimore in June 2023 and reported by the mainstream media.^9^ B, Hazard Mapping System data outlining the course of the smoke plume originating from provinces in Western Canada in June 2023, and migrating to the Eastern US, including Maryland (see eFigure in Supplement 1). C, Wildfire score according to census tract in Maryland counties included in the clinical analyses quantitating impact of wildfire smoke as defined by number of wildfire smoke days in June 2023 × wildfire smoke intensity (determined by satellite image opacity in each region). Regions of Maryland that designated in white background were not included in clinical analyses. D, An abrupt increase in PM_2.5_ concentration in the air during hotspot days was detected by the Environmental Protection Agency National Ambient Air Quality Standard and visually evident in Baltimore City.

### Cardiopulmonary Disease

The characteristics of the study cohort before standardizing for day of the week across years included a total of 2510 adult cardiopulmonary clinical encounters in June 2023 and 3774 adult cardiopulmonary clinical encounters in June 2018 and June 2019, respectively. After standardizing for calendar days across years, the number of cardiopulmonary clinical encounters in June 2023 was 2339 encounters (1098 female [46.9%]; 710 Black [30.4%], 1528 White [65.3%]) and for the combined 2018 and 2019 cohort was 3609 encounters (1690 female [46.8%]; 1181 Black [32.7%], 2269 White [62.9%]), respectively (Table 1). Patients with a cardiopulmonary clinical encounter during June 2023 were older (mean [SD] age, 68 [15] years vs 65 [15] years; *P* < .001) and reported a lower rate of heavy tobacco smoking compared with controls (276 of 1637 [16.9%] vs 547 of 2788 [19.6%]; *P* = .02).

**Table 1.** Clinical Profile of the Study Cohort

Characteristics	Patients, No. (%)		*P* value
	June 2018 plus June 2019 (n = 3609)	June 2023 (n = 2339)^a^	
Age, mean (SD), y	65 (15)	68 (15)	<.001
Sex			
Female	1690 (46.8)	1098 (46.9)	.57
Male	1919 (53.2)	1240 (53.0)	
Unknown or other	0	1 (<0.1)	
Race			
Asian	58 (1.6)	19 (0.8)	.004
Black or African American	1181 (32.7)	710 (30.4)	
White	2269 (62.9)	1528 (65.3)	
Other or unknown^b^	101 (2.8)	82 (3.5)	
BMI, mean (SD)	30 (12)	30 (8)	.21
Systemic hypertension	1505 (41.7)	955 (40.8)	.38
Coronary artery disease	1237 (34.3)	899 (38.4)	.002
Diabetes	1096 (30.4)	791 (33.8)	.009
Congestive heart failure	1020 (28.3)	821 (35.1)	<.001
Chronic obstructive pulmonary disease	923 (25.6)	595 (25.4)	.79
Obstructive sleep apnea	441 (12.2)	302 (12.9)	.51
Asthma	301 (8.5)	219 (9.4)	.20
Interstitial lung disease	94 (2.6)	77 (3.3)	.15
Connective tissue disease	56 (1.6)	39 (1.7)	.83
Liver cirrhosis	44 (1.2)	46 (2.0)	.03
Human immunodeficiency virus	41 (1.2)	27 (1.2)	>.99
Sickle cell disease	13 (0.4)	4 (0.2)	.27
Tobacco smoking status			
Never	1010 (30.1)	654 (31.2)	<.001
Former	1132 (33.7)	651 (31.0)	
Some days or light smoker	99 (2.9)	56 (2.7)	
Every day or heavy	547 (16.3)	276 (13.2)	
Smoker, current status unknown	568 (16.9)	460 (21.9)	
Social Vulnerability index, mean (SD)	0.43 (0.28)	0.40 (0.27)	<.001

Abbreviation: BMI, body mass index (calculated as weight in kilograms divided by height in meters squared).

^a^ Missingness for the June 2018 plus June 2019 control group and June 2023, respectively, was: BMI, 114 patients and 32 patients; clinical comorbidities, 56 patients and 21 patients; tobacco smoking status, 253 patients and 242 patients; Social Vulnerability Index, 251 patients and 158 patients.

^b^ Race was determined by patient self-report, documented by staff and extracted from the electronic health record. The categories of American Indian or Alaskan Native, Native American or other Pacific Islander, other, declined to answer, and unknown were combined into the other or unknown category.

After standardizing for calendar days across years, we observed 588 cardiopulmonary encounters occurred on hotspot days in June 2023, including 166 (28.2%) in ambulatory settings, 338 (57.5%) in inpatient settings, and 351 (59.7%) in emergency department settings. The proportion of patients with a clinical encounter in an ambulatory, inpatient, and emergency department setting relative to the total number of encounters in these settings during hotspot days was 166 of 533 (31.1%), 338 of 1455 (23.2%), and 351 of 1536 (22.9%), respectively (Table 2). Further data on the distribution of cardiopulmonary clinical encounters by setting are provided in Table 2; data on weekday vs weekend encounters and encounters stratified by cardiac non–heart failure, cardiac heart failure, and respiratory disease are provided in the supplement (eTable 3 and eTable 4 in Supplement 1).

**Table 2.** Settings for Clinical Encounters Occurring on Hotspot Days in 2023 and Matched Control Days in 2018 and 2019^a^

Characteristics	Patients, No. (%)					
	June 2023 (n = 2339)			June 2018 plus June 2019 (n = 3609)		
	Total, No.	Hotspot days	Non–hotspot days	Total, No.	Matched hotspot days	Matched non–hotspot days
All cardiopulmonary encounters	2339	588 (25.1)	1751 (74.9)	3609	806 (22.3)	2803 (77.7)
Emergency department	1536	351 (22.9)	1185 (77.1)	2266	461 (20.3)	1805 (79.7)
Inpatient	1455	338 (23.2)	1117 (76.8)	2368	508 (21.5)	1860 (78.5)
Ambulatory	533	166 (31.1)	367 (68.9)	697	183 (26.3)	514 (73.7)

^a^ Cardiopulmonary clinical encounter data are reported after standardizing for day of the week in 2018, 2019, and 2023 (using 4 full weeks of June in each year).

The proportion of cardiopulmonary clinical encounters that occurred on hotspot days was 588 of 2339 (25.1%) in June 2023 compared with 806 of 3609 (22.3%) during matched control days (*χ^2^* = 6.07; *P* = .01) (Table 2; eTable 5 in Supplement 1). After adjustment for covariates, the adjusted odds ratio (aOR) was 1.18 (95% CI, 1.03-1.34; *P* = .02). This finding was maintained after adjusting for covariates including day of the week (aOR, 1.17; 95% CI, 1.03-1.34; *P* = .02). When restricting the analysis to cardiac disease, there was a 20% increase in risk of a clinical encounter occurring on a hotspot day (aOR, 1.20; 95% CI, 1.01-1.42, *P* = .04) (Table 3). Among the cardiac disease cohort, the clinical encounter rate on hotspot days for patients with systematic hypertension was 26.8% in June 2023 compared with 22.4% during matched control days (*χ^2^* = 6.0; *P* = .01) and the adjusted odds of a clinical encounter occurring on a hotspot day was increased by 26% (aOR, 1.26; 95% CI, 1.03-1.54; *P* = .03). Significant differences in the rate of other highly prevalent cardiac subphenotypes, heart failure, or respiratory disease were not observed between groups.

**Table 3.** Probability of a Clinical Encounter on Hotspot Days in 2023 Compared With Matched Control Days in 2018 and 2019

Disease type^a^	Probability of clinical encounter, aOR (95% CI)^b^	*P* value
Cardiopulmonary	1.18 (1.03-1.34)	.02
Cardiac	1.20 (1.01-1.42)	.04

Abbreviation: aOR, adjusted odds ratio.

^a^ Cardiopulmonary disease refers to all cardiac non–heart failure, cardiac heart failure, and respiratory diseases included in this study; cardiac refers to cardiac non–heart failure. The *International Statistical Classification of Diseases and Related Health Problems, Tenth Revision (ICD-10)* codes for these phenotypes are provided in eTable 1 in Supplement 1.

^b^ Odds ratios were adjusted for age, sex, race, body mass index, smoking status, and overall social vulnerability index.

### Setting

There was a 55% increase in the adjusted risk for an outpatient clinical encounter on hotspot days (aOR, 1.55; 95% CI, 1.17-2.07; *P* = .003), whereas a significant difference between groups was not observed for emergency department or inpatient clinical encounters. There was no significant difference in the Area Deprivation Index (ADI) score for patients with a cardiopulmonary clinical encounter in June 2023 compared with June 2018 and 2019. However, patients with a cardiopulmonary encounter on hotspot days in June 2023 had greater socioeconomic advantage compared with patients with a cardiopulmonary encounter in June 2018 and 2019, as indicated by lower ADI score (mean [SD] score, 39.1 [21.1] vs 41.0 [23.7]; *P* = .05).

## Discussion

Recent evidence suggests that wildfire size and intensity are increasing in response to ongoing climate variability and change^13,14^; however, the detrimental health consequences of wildfire smoke at great distances from the original source are not well appreciated.^15^ Although wildfire smoke events in the Eastern US are rare,^16^ this study shows that large populations living on the US Eastern seaboard may nonetheless be vulnerable to adverse cardiopulmonary health events associated with wildfire smoke events emanating from Western or Central Canada, approximately 2100 miles distant. We leveraged the vast UMMS clinical operations network spanning 9 Maryland counties to identify a significant association between cardiopulmonary impairment and wildfire smoke, in contrast to prior reports of more limited geographic scope.^4^ In this regard, we were able to report adverse clinical events in residents of diverse urban (Baltimore and Prince George’s Counties) and rural (Eastern Shore) communities due to transcontinental migration of EPA-defined air pollutants.^17^

Although clinical encounters were increased in association with wildfire smoke across each of the settings we analyzed, our findings suggest that ambulatory care was particularly susceptible to change in patient volume during hotspot days. This may reflect a modest elevation in cardiopulmonary disease burden among many patients commensurate with the decaying wildfire smoke plume intensity over its 3400 km transcontinental trajectory, possibly in combination with appropriate engagement with acute care centers by the study cohort; or, conversely, the volatility particular to ambulatory care indicates challenges related to accessing tertiary referral centers among at-risk patients. It is possible that patients pursuing ambulatory-level care were responding to changes in self-monitored blood pressure or symptoms related to hypertension, because hypertension emerged as an important cardiac subphenotype associated with elevated clinical encounters on hotspot days. The relevance of hypertension for anticipating clinical response to wildfire smoke is not well-defined,^18^ and, thus, this observation exposes a novel comorbidity that may be important to consider for risk stratifying patients when air quality conditions deteriorate.

The potential adverse health events associated with climate change–driven wildfire smoke episodes reported here are important for several reasons. First, recognizing cardiopulmonary risk to general populations distant from the origin of wildfire smoke events is obviously relevant to public health and other physicians. Second, strategies that integrate up-to-date atmospheric data into electronic health record systems could help individualize risk assessment based on patient clinical profile and residence location. Third, our findings raise the prospect of designing location-specific early warnings and therapeutic interventions to prospectively mitigate the adverse health effects associated with wildfire smoke air pollution. However, managing the acute population health changes associated with exposure to distant wildfire smoke is likely to require engagement from clinicians, climate experts, and government officials. In response to the 2023 Canadian wildfires, the Maine Department of Environmental Protection reconfigured their PM_2.5_ monitoring system to improve forecasting adverse changes to regional air quality from wildfire smoke,^19^ for example, thereby setting the stage for further collaborations integrating atmospheric monitoring with public health initiatives.

Our study cohort was diverse across geo-sociodemographic parameters and comprised a sizeable representation among Black and African American patients. Socioeconomic disadvantage, which has been reported for regions similar to areas included in this study,^20^ is associated with increased prevalence of cardiopulmonary disease^21^ and a susceptibility to wildfire smoke.^22,23^ Our observation that adverse cardiopulmonary clinical encounters were more frequent in those patients with socioeconomic advantages may underscore the importance of health care access, ie, Maryland patients with socioeconomic disadvantage may have been undertreated due to lesser access to care. Yu and colleagues^24^ observed recently that high pollution days in Pennsylvania from the 2023 Canadian wildfire smoke plume, which corresponded to hotspot days in our study, were associated with less human mobility. These data corroborate with our findings that suggest complex interaction between socioeconomic and geographic characteristics contribute to the consequences of distant wildfire smoke on human health.

### Limitations

This study had limitations. Maryland was not the only Eastern US state affected, and other study limitations include biases inherent to retrospective studies using *ICD-10* codes and alternative causes of cardiopulmonary events that were unmeasured. While wildfires are known to produce countless number of air pollutants, our study focused specifically on wildfire-related PM_2.5_. Future studies need to consider other wildfire-related pollutants as well as the interactive effect of these co-pollutants. This study was limited by biases inherent in a retrospective study design, and our approach did not capture information on the severity of comorbidities or clinical parameters during encounters. This information, in turn, would be helpful for profiling the clinical association of the wildfire smoke plume on patients in this study.

## Conclusions

In this case-only study of a large medical system, cardiopulmonary disease burden was increased for residents of Maryland. This observation was likely associated with contemporaneous wildfire smoke–based infiltration of polluted smoke air originating from Western Canada, up to 2100 miles remotely. Taken together, these data suggest that large and highly populous geographic regions not traditionally regarded as susceptible to the adverse effects of wildfire smoke, such as in the Eastern US, may nonetheless experience adverse health consequences associated with smoke from wildfires originating from remote distances. These data support future prospective studies that advance anticipatory care measures for cardiopulmonary disease in association with the acute deterioration of air quality due to smoke from intense wildfires.

## Notes

Supplement 1.
eMethods.

eReferences
**eTable 1.** *ICD-10* Codes Used in This Study for Each of the Three Clinical Phenotype Categories **eTable 2.** Proportion of Missingness by Diagnosis in the Study Group **eTable 3.** Distribution of Cardiopulmonary Clinical Encounters Stratified by Setting **eTable 4.** Frequency of Diagnoses for Patients in the Analysis **eTable 5.** Clinical Profile of Patients With a Cardiopulmonary Encounter During a Hotspot Day in 2023 vs Patients With a Cardiopulmonary Encounter During an Orthologous Hotspot Day in 2018-2019

Supplement 2.
Data Sharing Statement
