# Supplementary material for: Polluted Air from Canadian Wildfires and Cardiopulmonary Disease in the Eastern US
Source: JAMA Netw Open. 2024 Dec 13;7(12):e2450759. doi: 10.1001/jamanetworkopen.2024.50759 (PMC11645649; doi:10.1001/jamanetworkopen.2024.50759)
Supplement: Supplement 1. — eMethods. eReferences eTable 1. ICD-10 Codes Used in This Study for Each of the Three Clinical Phenotype Categories eTable 2. Proportion of Missingness by Diagnosis in the Study Group eTable 3. Distribution of Cardiopulmonary Clinical Encounters Stratified by Setting eTable 4. Frequency of Diagnoses for Patients in the Analysis eTable 5. Clinical Profile of Patients With a Cardiopulmonary Encounter During a Hotspot Day in 2023 vs Patients With a Cardiopulmonary Encounter During an Orthologous Hotspot Day in 2018-2019 [file jamanetwopen-e2450759-s001.pdf]

Supplemental Online Content

Maldarelli ME, Song H, Brown CH, et al. Polluted air from Canadian Wildfires and Cardiopulmonary Disease in the Eastern US. *JAMA Netw Open.* 2024;7(12):e2450759. doi:10.1001/jamanetworkopen.2024.50759

eMethods

eReferences

eTable 1. *ICD-10* Codes Used in This Study for Each of the Three Clinical Phenotype Categories

eTable 2. Proportion of Missingness by Diagnosis in the Study Group

eTable 3. Distribution of Cardiopulmonary Clinical Encounters Stratified by Setting

eTable 4. Frequency of Diagnoses for Patients in the Analysis

eTable 5. Clinical Profile of Patients With a Cardiopulmonary Encounter During a Hotspot Day in 2023 vs Patients With a Cardiopulmonary Encounter During an Orthologous Hotspot Day in 2018-2019

This supplemental material has been provided by the authors to give readers additional information about their work.

© 2024 Maldarelli ME et al. *JAMA Network Open.*

Methods

Estimating wildfire-related $PM_{2.5}$. To determine whether the census tract was under wildfire smoke plume on a given day, we utilized National Oceanic and Atmospheric Administration's (NOAA) Hazard Mapping System (HMS) data. The HMS utilizes satellite observations to categorize the density of smoke within each polygon as light, medium, and heavy based on satellite image opacity.[1-3] To calculate wildfire related $PM_{2.5}$, each census tract was first assigned daily $PM_{2.5}$ concentration data from nearest U.S. EPA Air Quality System (AQS) $PM_{2.5}$ monitor. If a census tract was not under a wildfire smoke plume polygon, the estimated wildfire-related $PM_{2.5}$ was assigned a value 0 $\mu g/m^3$, and non-wildfire-related $PM_{2.5}$ was assigned the daily $PM_{2.5}$ concentrations. If a census tract was under a wildfire smoke plume, the wildfire-related $PM_{2.5}$ concentration for that date was computed by subtracting the seasonal non-smoke background $PM_{2.5}$ concentration from the $PM_{2.5}$ concentration for that given day. Seasonal background $PM_{2.5}$ concentrations represent median $PM_{2.5}$ concentrations in the absence of wildfire smoke plume during the summer months (June-August). When a smoke plume was detected but the $PM_{2.5}$ concentration was less than the seasonal background $PM_{2.5}$, a value of 0 $\mu g/m^3$ was assigned for the wildfire-related $PM_{2.5}$. Non-wildfire-related $PM_{2.5}$ was determined by subtracting the wildfire-related $PM_{2.5}$ from the $PM_{2.5}$ concentration.

Smoke plumes. Smoke plumes were retrieved from NOAA's HMS for June 7, 2023 showing the trajectory from Canadian wildfires southeast into the northeast and mid-Atlantic United States. This smoke was driven by a high-pressure zone known as an omega block. Wildfire detections (which are displayed as red dots in Figure 1) were retrieved from NASA's MODIS sensor for the same date (only Canadian hot spots are displayed).

© 2024 Maldarelli ME et al. *JAMA Network Open.*

Co-variates for multi-variable logistic regression. Patients with >1 of cardiac-nonheart failure, cardiac-heart failure, and respiratory diagnoses at the time of a clinical encounter were tabulated once for cardiopulmonary disease and once for each corresponding phenotype in subgroup analyses. Clinical characteristics abstracted included age, sex, race, body mass index, systemic hypertension, diabetes, coronary artery disease, congestive heart failure, chronic obstructive pulmonary disease, interstitial lung disease, obstructive sleep apnea, human immunodeficiency virus, sickle cell disease, connective tissue disease, liver cirrhosis, asthma, smoking status, national Area Deprivation Index (ADI), state ADI, and social vulnerability index (SVI). Patient addresses were geocoded to extract relevant ADI and SVI scores using Geocodio™. In the adjusted models, the covariates are: Age, sex (male, female), race (Black or African American, White, Asian, other or unknown), smoking status (Every day/heavy user, some days/light smoker, smoker but current status unknown, former smoker, status unknown), body mass index, and social vulnerability index. For the model with day of the week it was only possible to adjust for days of the week in which hotspot days occurred: Tuesday, Wednesday, Thursday, Friday.

© 2024 Maldarelli ME et al. *JAMA Network Open.*

References

1. Office of Satellite and Product Operations - Hazard Mapping System. Accessed May 18, 2022. https://www.ospo.noaa.gov/Products/land/hms.html#about
2. Ruminski M, Draxler R, Kondragunta S, Zeng J. Recent Changes to the Hazard Mapping System. Published online 2006.
3. Schroeder W, Ruminski M, Csiszar I, et al. Validation analyses of an operational fire monitoring product: The Hazard Mapping System. Int J Remote Sens. 2008;29(20):6059-6066. doi:10.1080/01431160802235845

© 2024 Maldarelli ME et al. *JAMA Network Open.*

ICD-10 Codes						
Cardiac-non heart failure			Cardiac heart failure	Respiratory		
I00	I25112	I341	I501	J0100	J441	J704
I010	I25118	I342	I5020	J0101	J4481	J705
I011	I25119	I348	I5021	J0110	J4489	J708
I012	I252	I3481	I5022	J0111	J449	J709
I018	I253	I3489	I5023	J0120	J4520	J80
I019	I2541	I349	I5030	J0121	J4521	J810
I020	I2542	I350	I5031	J0130	J4522	J811
I029	I255	I351	I5032	J0131	J4530	J82
I050	I256	I352	I5033	J0140	J4531	J8281
I051	I25700	I358	I5040	J0141	J4532	J8282
I052	I25701	I359	I5041	J0180	J4540	J8283
I058	I25702	I360	I5042	J0181	J4541	J8289
I059	I25708	I361	I5043	J0190	J4542	J8409
I060	I25709	I362	I50810	J0191	J4550	J8410
I061	I25710	I368	I50811	J028	J4551	J84111
I062	I25711	I369	I50812	J029	J4552	J84112
I068	I25712	I370	I50813	J040	J45901	J84113
I069	I25718	I371	I50814	J0410	J45902	J84114
I070	I25719	I372	I5082	J0411	J45909	J84115
I071	I25720	I378	I5083	J042	J45990	J84116
I072	I25721	I379	I5084	J0430	J45991	J84117
I078	I25722	I38	I5089	J0431	J45998	J8417
I079	I25728	I39	I509	J050	J470	J84170
I080	I25729	I400		J0510	J471	J84178
I081	I25730	I401		J0511	J479	J8489
I082	I25731	I408		J060	J4A0	J849
I083	I25732	I409		J208	J4A8	J90
I088	I25738	I41		J209	J4A9	J910
I089	I25739	I420		J219	J60	J918
I090	I25750	I421		J300	J61	J920
I091	I25751	I422		J301	J620	J929
I092	I25752	I423		J302	J628	J930
I0981	I25758	I424		J305	J630	J9311
I0989	I25759	I425		J3081	J631	J9312
I099	I25760	I426		J3089	J632	J9381
I10	I25761	I427		J309	J633	J9382
I110	I25762	I428		J310	J634	J9383
I119	I25768	I429		J311	J635	J939
I120	I25769	I43		J312	J636	J940
I129	I25790	I440		J320	J64	J941
I130	I25791	I441		J321	J65	J942
I1310	I25792	I442		J322	J660	J948
I1311	I25798	I4430		J323	J661	J949
I132	I25799	I4439		J324	J662	J9600
I150	I25810	I444		J328	J668	J9601
I151	I25811	I445		J329	J670	J9602
I152	I25812	I4460		J3489	J671	J9610
I158	I2582	I4469		J349	J672	J9611
I159	I2583	I447		J383	J673	J9612
I160	I2584	I450		J384	J674	J9620
I161	I2585	I4510		J385	J675	J9621
I169	I2589	I4519		J386	J676	J9622
I1A0	I259	I452		J387	J677	J9690
I200	I2601	I453		J390	J678	J9691
I201	I2602	I454		J391	J679	J9692
I202	I2609	I455		J392	J680	J9801
I208	I2690	I456		J393	J681	J9809

© 2024 Maldarelli ME et al. *JAMA Network Open.*

I2081	I2692	I4581		J398	J682	J9811
I2089	I2693	I4589		J399	J683	J9819
I209	I2694	I459		J40	J684	J982
I2101	I2699	I462		J410	J688	J983
I2102	I270	I468		J411	J689	J984
I2109	I271	I469		J418	J690	J985
I2111	I272	I470		J42	J691	J9851
I2119	I2720	I471		J430	J698	J9859
I2121	I2721	I4710		J431	J700	J986
I2129	I2722	I4711		J432	J701	J988
I213	I2723	I4719		J438	J702	J989
I214	I2724	I472		J439	J703	J99
I219	I2729	I4720		J440		
I21A1	I2781	I4721				
I21A9	I2782	I4729				
I21B	I2783	I479				
I220	I2789	I480				
I221	I279	I481				
I222	I280	I4811				
I228	I281	I4819				
I229	I288	I482				
I230	I289	I4820				
I231	I300	I4821				
I232	I301	I483				
I233	I308	I484				
I234	I309	I4891				
I235	I310	I4892				
I236	I311	I4901				
I237	I312	I4902				
I238	I313	I491				
I240	I3131	I492				
I241	I3139	I493				
I248	I314	I4940				
I2481	I318	I4949				
I2489	I319	I495				
I249	I32	I498				
I2510	I330	I499				
I25110	I339	I52				
I25111	I340	I5A				

eTable 1. ICD-10 codes used in this study for each of the three clinical phenotype categories.

© 2024 Maldarelli ME et al. *JAMA Network Open.*

Diagnosis	All Cases	Complete Cases	Incomplete Cases	% Incomplete
Cardiac<br>(non-Heart Failure)	3348	2977	371	11.1
Heart Failure	792	728	64	8.1
Respiratory	2231	256	175	7.8

eTable 2. Proportion of missingness by diagnosis in the study group. Incomplete cases were driven largely by missingness for body mass index (BMI) and social vulnerability index (SVI).

© 2024 Maldarelli ME et al. *JAMA Network Open.*

Cardiopulmonary Encounters						
	**Not hotspot**			**Hotspot**		
Year	Total	Weekday	Weekend	Total	Weekday	Weekend
2018-2019	2803	2124	679	806	806	0
2023	1751	1292	459	588	588	0
**Setting**						
**Emergency Room**						
	**Not Hotspot**			**Hotspot**		
	Total	Weekday	Weekend	Total	Weekday	Weekend
2018-2019	1805	1214	591	461	461	0
2023	1185	770	415	351	351	0
**Inpatient**						
	**Not Hotspot**			**Hotspot**		
	Total	Weekday	Weekend	Total	Weekday	Weekend
2018-2019	1860	1323	537	508	508	0
2023	1117	748	369	338	338	0
**Ambulatory**						
	**Not Hotspot**			**Hotspot**		
	Total	Weekday	Weekend	Total	Weekday	Weekend
2018-2019	514	504	10	183	183	0
2023	367	354	13	166	166	0

eTable 3. Distribution of cardiopulmonary clinical encounters stratified by setting. Non-hotspot days represent matched days in June 2018+2019 that correspond to hotspot days from 2023.

© 2024 Maldarelli ME et al. *JAMA Network Open.*

Cardiac (non-Heart Failure)	Heart Failure	Respiratory	N
1	0	0	3037
0	0	1	1964
0	1	0	555
1	0	1	155
1	1	0	125
0	1	1	81
1	1	1	31
			5948

eTable 4. Frequency of diagnoses for patients in the analysis.

© 2024 Maldarelli ME et al. *JAMA Network Open.*

	N	June 2018-2019<br>N=806	June 2023<br>N=588	P-value
**Age (yr)**	1,394	66 (15)	67 (14)	0.06
**Sex**	1,394			0.28
Female		372 (46%)	289 (49%)	
Male		434 (54%)	299 (51%)	
Unknown or other		0 (0%)	0 (0%)	
**Race**	1,394			0.07
White		515 (64%)	390 (66%)	
Black or African American		234 (29%)	174 (30%)	
Asian		23 (2.9%)	6 (1%)	
Other or Unknown		34 (4.2%)	18 (3.1%)	
**BMI (kg/m^2)**	1,353	30 (14)	30 (8)	0.71
**Tobacco smoking status**	1,282			<0.001
Never		238 (32%)	169 (32%)	
Former		242 (32%)	147 (28%)	
Some Days/Light Smoker		21 (2.8%)	9 (1.7%)	
Every Day/Heavy		109 (15%)	58 (11%)	0.30
Smoker, Current Status Unknown		140 (19%)	149 (28%)	
**Social Vulnerability Index**	1,289	0.43 (0.29)	0.41 (0.26)	0.10
**Day of the week of a clinical encounter**	1,394			
Tuesday		150 (19%)	104 (18%)	0.66
Wednesday		279 (35%)	215 (37%)	0.45
Thursday		240 (30%)	177 (30%)	0.90
Friday		137 (17%)	92 (16%)	0.50

eTable 5. Clinical profile of patients with a cardiopulmonary encounter during a hotspot day in 2023 vs. patients with a cardiopulmonary encounter during an orthologous hot-spot day in 2018-2019.

© 2024 Maldarelli ME et al. *JAMA Network Open.*

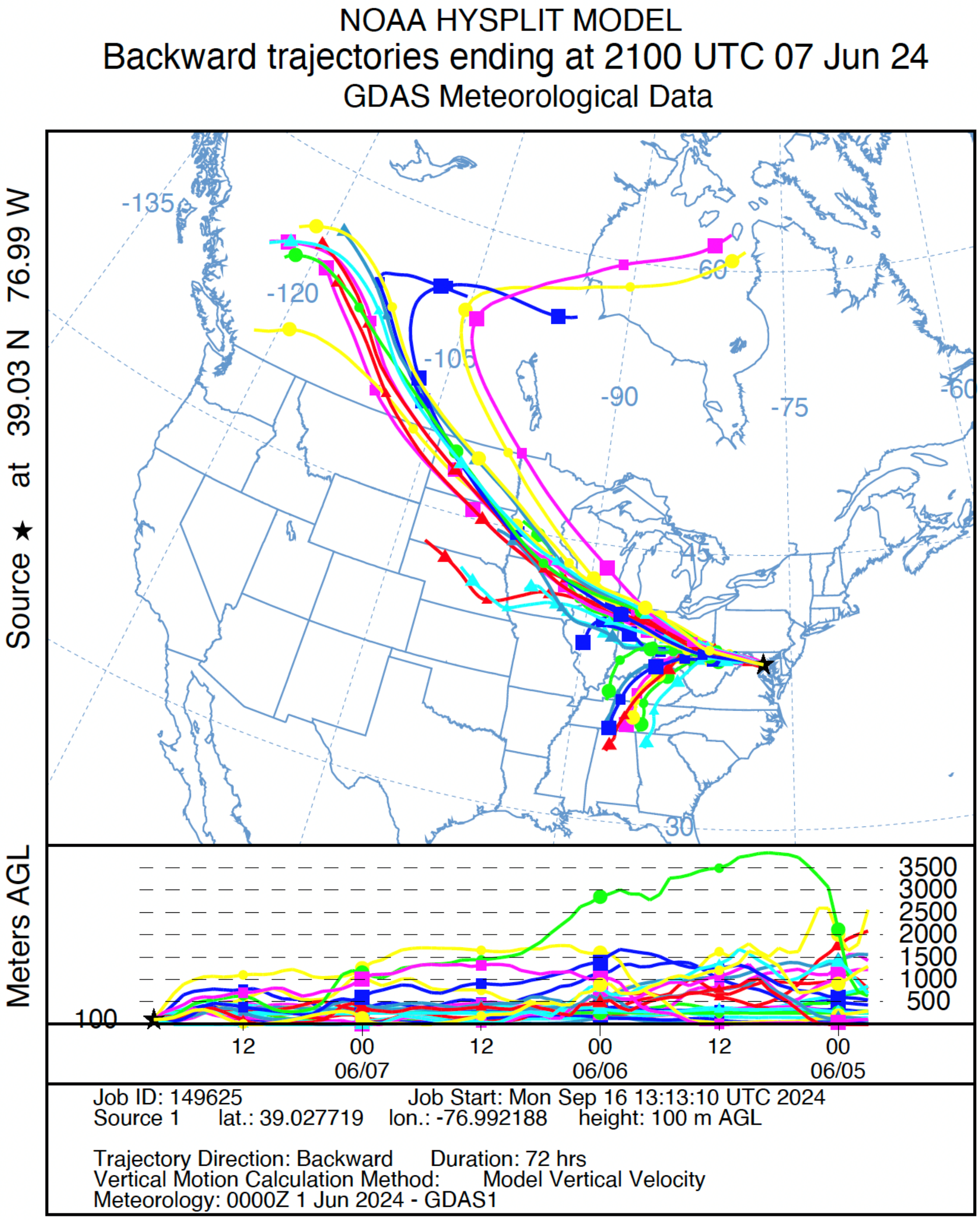

eFigure 1

© 2024 Maldarelli ME et al. *JAMA Network Open.*

eFigure Legend

eFigure 1. Backward trajectory of air masses above Baltimore City at 21:00 UTC, 7th June 2023. The figure shows backward trajectory of air mass spanning 72 hours prior to 7th June 2023.

© 2024 Maldarelli ME et al. *JAMA Network Open.*
