# Supplementary material for: Polluted Air from Canadian Wildfires and Cardiopulmonary Disease in the Eastern US
Source: JAMA Netw Open. 2024 Dec 13;7(12):e2450759. doi: 10.1001/jamanetworkopen.2024.50759 (PMC11645649; doi:10.1001/jamanetworkopen.2024.50759)
Supplement: Supplement 2. — Data Sharing Statement [file jamanetwopen-e2450759-s002.pdf]

Data Sharing Statement

Maldarelli. Polluted Air from Canadian Wildfires and Cardiopulmonary Disease in the Eastern US. *JAMA Netw Open*. Published December 13, 2024.
doi:10.1001/jamanetworkopen.2024.50759

Data
Data available: No

Additional Information
Explanation for why data not available: Patient-level data may be made available to approved investigators under a DUA with the University of Maryland Medical System and IRB approval.
